# Weighted Multilinear Hardy Operators on Herz Type Spaces

**DOI:** 10.1155/2014/420408

**Published:** 2014-01-05

**Authors:** Shuli Gong, Zunwei Fu, Bolin Ma

**Affiliations:** ^1^College of Mathematics and Econometrics, Hunan University, Changsha, Hunan 410082, China; ^2^Department of Mathematics, Linyi University, Linyi, Shandong 276005, China; ^3^College of Mathematics Physics and Information Engineering, Jiaxing University, Jiaxing, Zhejiang 314001, China

## Abstract

This paper focuses on the bounds of weighted multilinear Hardy operators on the product Herz spaces and the product Morrey-Herz spaces, respectively. We present a sufficient condition on the weight function that guarantees weighted multilinear Hardy operators to be bounded on the product Herz spaces. And the condition is necessary under certain assumptions. Finally, we extend the obtained results to the product Morrey-Herz spaces.

## 1. Introduction

Let *f* be a nonnegative integral function on ℝ^+^. The Hardy operator *H* is defined by *Hf*(*x*) = (1/*x*)∫_0_^*x*^ *f*(*t*)*dt*, *x* > 0. For the operator *H*, Hardy et al. [1] proved that the inequality

(1) $$\begin{array}{c} {||Hf||}_{L^{p}}\leq \frac{p}{p-1}{||f||}_{L^{p}} \end{array}$$

holds, where 1 < *p* < *∞* and the constant *p*/(*p* − 1) is the best possible. Usually, we call (1) classical Hardy's inequality. With the development of analysis theory, many types of Hardy's inequalities have been discussed. For example, a quite number of papers dealt with the various generalizations, numerous variants, and applications of Hardy's inequalities in the past few years. On the detailed discussions of Hardy's inequalities, we choose to refer to [2, 3].

In 1984, Carton-Lebrun and Fosset [4] gave the definition of the weighted Hardy operator *H*_*ω*_ to be

(2) $$\begin{array}{c} H_{\omega }f\left(x\right)∶=\overset{1}{\underset{0}{\int }}f\left(tx\right)\omega \left(t\right)dt,\;x\in {\mathbb{R}}^{n}, \end{array}$$

where *ω* : [0,1]→[0, *∞*) is a measurable function and *f* is a complex-valued measurable function on ℝ^*n*^. It is obvious that *H*_*ω*_ degenerates into the classical Hardy operator *H* when *ω* ≡ 1, *n* = 1, and *f* is defined on ℝ^+^. In addition, we call the adjoint operator of *H*_*ω*_ the weighted Cesàro average *G*_*ω*_ *f*. And the definition of *G*_*ω*_ *f* is

(3) $$\begin{array}{c} \left(G_{\omega }f\right)\left(x\right)=\overset{1}{\underset{0}{\int }}f\left(\frac{x}{t}\right)t^{-n}\omega \left(t\right)dt. \end{array}$$

When *ω* ≡ 1 and *n* = 1, *G*_*ω*_ becomes the classical Cesàro operator *G*

(4) $$\begin{array}{c} Gf\left(x\right)=\{\begin{array}{cc} \overset{\infty }{\underset{x}{\int }}\frac{f\left(y\right)}{y}dy, & x>0,\\ -\overset{x}{\underset{-\infty }{\int }}\frac{f\left(y\right)}{y}dy, & x<0. \end{array} \end{array}$$

It is easy to get that *H*_*ω*_ and *G*_*ω*_ satisfy

(5) $$\begin{array}{c} \int_{{\mathbb{R}}^{n}}\left(H_{\omega }f\right)\left(x\right)g\left(x\right)dx=\int_{{\mathbb{R}}^{n}}f\left(x\right)\left(G_{\omega }g\right)\left(x\right)dx, \end{array}$$

when *f* ∈ *L* ^*p*^(ℝ^*n*^), *g* ∈ *L* ^*q*^(ℝ^*n*^), 1 < *p* < *∞*, 1/*p* + 1/*q* = 1. This means that *H*_*ω*_ and *G*_*ω*_ satisfy the commutative rule *H*_*ω*_ *G*_*ω*_ = *G*_*ω*_ *H*_*ω*_.

Under certain conditions on *ω*, Carton-Lebrun and Fosset [4] proved that *H*_*ω*_ maps *L* ^*p*^(ℝ^*n*^) into itself for 1 < *p* < *∞*. They also pointed out that the operator *H*_*ω*_ commutes with the Hilbert transform when *n* = 1 and with certain Calderón-Zygmund singular integral operators including the Riesz transform when *n* ≥ 2. Refer to Xiao [5] for the further extension of the results above; see also [6, 7].

Since Herz space is a natural generalization of weighted Lebesgue spaces with power weights, researchers are also interested in studying the boundedness of *H*_*ω*_ on Herz spaces. To make the description more clear below, we review the definition of the Herz spaces now. In the following definitions, *χ*_*k*_ = *χ*_*C*_*k*__, *C*_*k*_ = *B*_*k*_∖*B*_*k*−1_, and *B*_*k*_ = {*x* ∈ ℝ^*n*^ : |*x* | ≤2^*k*^}, for *k* ∈ *ℤ*, ${\tilde{C}}_{k}=C_{k}$, ${\tilde{C}}_{0}=B_{0}$, and ${\tilde{\chi }}_{k}=\chi_{C_{k}}$, for *k* ∈ *ℕ*, and *χ*_*E*_ is the characteristic function of a set *E*.

**Definition 1 (see [8])**

Let *α* ∈ ℝ, 0 < *p* ≤ *∞*, 0 < *q* < *∞*.

(1) The homogeneous Herz spaces ${\dot{K}}_{q}^{\alpha ,p}({\mathbb{R}}^{n})$ is defined by

(6) $$\begin{array}{c} {\dot{K}}_{q}^{\alpha ,p}\left({\mathbb{R}}^{n}\right)=\left\{f\in L_{\mathrm{loc}}^{q}\left({\mathbb{R}}^{n}\setminus \left\{0\right\}\right):{||f||}_{{\dot{K}}_{q}^{\alpha ,p}({\mathbb{R}}^{n})}<\infty \right\}, \end{array}$$

where

(7) $$\begin{array}{c} {||f||}_{{\dot{K}}_{q}^{\alpha ,p}({\mathbb{R}}^{n})}={\left\{\overset{\infty }{\underset{k=-\infty }{\sum }}2^{k\alpha p}{||f\chi_{k}||}_{L^{q}\left({\mathbb{R}}^{n}\right)}^{p}\right\}}^{1/p}. \end{array}$$

(2) The inhomogenous Herz spaces *K*_*q*_^*α*,*p*^(ℝ^*n*^) is defined by

(8) $$\begin{array}{c} K_{q}^{\alpha ,p}\left({\mathbb{R}}^{n}\right)=\left\{f\in L_{\mathrm{loc}}^{q}\left({\mathbb{R}}^{n}\right):{||f||}_{K_{q}^{\alpha ,p}({\mathbb{R}}^{n})}<\infty \right\}, \end{array}$$

where

(9) $$\begin{array}{c} {||f||}_{K_{q}^{\alpha ,p}({\mathbb{R}}^{n})}={\left\{\overset{\infty }{\underset{k=0}{\sum }}2^{k\alpha p}{||f{\tilde{\chi }}_{k}||}_{L^{q}\left({\mathbb{R}}^{n}\right)}^{p}\right\}}^{1/p}, \end{array}$$

with usual modifications made when *p* = *∞* or *q* = *∞*.

In [9], Wu gave the definition of Morrey-Herz spaces.

**Definition 2**

Let *α* ∈ ℝ, 0 < *p* ≤ *∞*, 0 < *q* < *∞*, and *λ* ≥ 0.

(1) The homogeneous Morrey-Herz space $M{\dot{K}}_{p,q}^{\alpha ,\lambda }({\mathbb{R}}^{n})$ is defined by

(10) $$\begin{array}{c} M{\dot{K}}_{p,q}^{\alpha ,\lambda }\left({\mathbb{R}}^{n}\right)∶=\left\{f\in L_{\mathrm{loc}}^{q}\left({\mathbb{R}}^{n}\setminus \left\{0\right\}\right):{||f||}_{M{\dot{K}}_{p,q}^{\alpha ,\lambda }({\mathbb{R}}^{n})}<\infty \right\}, \end{array}$$

where

(11) $$\begin{array}{c} {||f||}_{M{\dot{K}}_{p,q}^{\alpha ,\lambda }({\mathbb{R}}^{n})}∶=\underset{k_{0}\in \mathbb{Z}}{\sup}2^{-k_{0}\lambda }{\left\{\overset{k_{0}}{\underset{k=-\infty }{\sum }}2^{k\alpha p}{||f\chi_{k}||}_{L^{q}({\mathbb{R}}^{n})}^{p}\right\}}^{1/p}. \end{array}$$

(2) The inhomogeneous Morrey-Herz space *MK*_*p*,*q*_^*α*,*λ*^(ℝ^*n*^) is defined by

(12) $$\begin{array}{c} MK_{p,q}^{\alpha ,\lambda }\left({\mathbb{R}}^{n}\right)∶=\left\{f\in L_{\mathrm{loc}}^{q}\left({\mathbb{R}}^{n}\right):{||f||}_{MK_{p,q}^{\alpha ,\lambda }({\mathbb{R}}^{n})}<\infty \right\}, \end{array}$$

where

(13) $$\begin{array}{c} {||f||}_{MK_{p,q}^{\alpha ,\lambda }({\mathbb{R}}^{n})}∶=\underset{k_{0}\in \mathbb{N}}{\sup}2^{-k_{0}\lambda }{\left\{\overset{k_{0}}{\underset{k=0}{\sum }}2^{k\alpha p}{||f{\tilde{\chi }}_{k}||}_{L^{q}\left({\mathbb{R}}^{n}\right)}^{p}\right\}}^{1/p}, \end{array}$$

with usual modifications made when *p* = *∞* or *q* = *∞*.

From the above definitions, it is not difficult to note that ${\dot{K}}_{p}^{0,p}({\mathbb{R}}^{n})\;=$ *K*_*p*_^0,*p*^(ℝ^*n*^)  = $L^{p}({\mathbb{R}}^{n}),{\dot{K}}_{p}^{\alpha /p,p}({\mathbb{R}}^{n})=K_{p}^{\alpha /p,p}({\mathbb{R}}^{n})=L^{p}(|x{|}^{\alpha }dx)$ for all 0 < *p* ≤ *∞* and *α* ∈ ℝ. Moreover, the homogenous Herz spaces ${\dot{K}}_{q}^{\alpha ,p}({\mathbb{R}}^{n})$, the homogenous Morrey-Herz spaces $M{\dot{K}}_{p,q}^{\alpha ,\lambda }({\mathbb{R}}^{n})$, and the Morrey spaces *M* ^*q*,*λ*^(ℝ^*n*^) (see [10, 11]) satisfy $M{\dot{K}}_{p,q}^{\alpha ,0}({\mathbb{R}}^{n})={\dot{K}}_{q}^{\alpha ,p}({\mathbb{R}}^{n}),M^{q,\lambda }({\mathbb{R}}^{n})\subset M{\dot{K}}_{q,q}^{0,\lambda }({\mathbb{R}}^{n})$. In [12], Liu and Fu discussed the boundedness of the weighted Hardy operator *H*_*ω*_ on the Herz space ${\dot{K}}_{q}^{\alpha ,p}({\mathbb{R}}^{n})$. Conditions on the weighted function *ω* were presented to guarantee that *H*_*ω*_ is bounded on ${\dot{K}}_{q}^{\alpha ,p}({\mathbb{R}}^{n})$. They also estimated the corresponding operator norm. And in [13], Fu and Lu further extended the results of [12] to the Morrey-Herz space $M{\dot{K}}_{p,q}^{\alpha ,\lambda }({\mathbb{R}}^{n})$.

In the past few years, the properties of multilinear operators have also been extensively studied by researchers. There are two reasons for this. First, the multilinear operators are the generalization of the linear ones, and its study makes the research contents of analysis theory more rich. Second, the multilinear operators naturally appear in analysis. The study of multilinear operators is traced to the multilinear singular integral operator theory (see [14]). For more detailed studies on multilinear operators, the readers refer to [15–18] and the references therein. Recently, we have studied the boundedness of weighted multilinear Hardy operators *ℋ*_*ω*_^*m*^ on the product of Lebesgue spaces and central Morrey spaces in [19]. Based on these results and inspired by the results of [12, 13], this paper further concerns the boundedness of *ℋ*_*ω*_^*m*^ on the product Herz spaces and the product Morrey-Herz spaces. We first recall the definition of *ℋ*_*ω*_^*m*^.

**Definition 3**

Let *m* ∈ *ℕ*, *m* ≥ 2, and

(14) $$\begin{array}{c} \omega :\;\overset{m\;\text{times}}{\overset{︷}{\left[0,1\right]\times \left[0,1\right]\times \cdots \times \left[0,1\right]}}⟶\left[0,\infty \right) \end{array}$$

be an integrable function. The *weighted multilinear Hardy operator ℋ*_*ω*_^*m*^ is defined by

(15) ℋωm(f→)(x)∶=∫0<t1,t2,…,tm<1(∏i=1mfi(tix))ω(t→)dt→,  x∈ℝn,

where $\vec{f}∶=(f_{1},\ldots ,f_{m}),\omega (\vec{t})∶=\omega (t_{1},t_{2},\ldots ,t_{m}),d\vec{t}:=dt_{1}\cdots dt_{m}$, and *f*_*i*_ (*i* = 1,…, *m*) are complex-valued measurable functions on ℝ^*n*^. When *m* = 2, *ℋ*_*ω*_^*m*^ is referred to as bilinear.

In accordance with the case of *H*_*ω*_, we also recall the definition of the weighted multilinear Cesàro operator *𝒢*_*ω*_ that is the adjoint operator of *ℋ*_*ω*_^*m*^.

**Definition 4**

Let *m* ∈ *ℕ*, *m* ≥ 2, and *ω* : [0,1]×[0,1]^*m*−1^ → [0, *∞*) be an integrable function. The weighted multilinear Cesàro operator *𝒢*_*ω*_^*m*^ is defined by

(16) 𝒢ωm(f→)(x)=∫0<t1,t2,…,tm<1(∏i=1mfi(xti)(ti)−n)ω(t→)dt→,  x∈ℝn,

where *f*_*i*_ are measurable complex-valued functions on ℝ^*n*^, 1 ≤ *i* ≤ *m*.

Note that *ℋ*_*ω*_^*m*^ and *𝒢*_*ω*_^*m*^ do not satisfy the following commutative rule:

(17) $$\begin{array}{c} \int_{{\mathbb{R}}^{n}}\left({ℋ}_{\omega }^{m}f\right)\left(x\right)g\left(x\right)dx=\int_{{\mathbb{R}}^{n}}f\left(x\right)\left({𝒢}_{\omega }^{m}g\right)\left(x\right)dx. \end{array}$$

This is different from the case of *H*_*ω*_ and *G*_*ω*_.

The paper is organized as follows. In Section 2, we present the estimate of the boundedness of *ℋ*_*ω*_^*m*^ on the product Herz spaces. In Section 3, we give the estimates of the boundedness of *ℋ*_*ω*_^*m*^ on the product Morrey-Herz spaces.

## 2. Boundedness of *ℋ*_*ω*_^*m*^ on the Product of Herz Spaces

We give the first main result of this paper.

**Theorem 5**

Let *α*, *α*_1_,…, *α*_2_ ∈ ℝ, 1 < *p*, *p*_*i*_, *q*, *q*_*i*_ < *∞* and *α*_1_ + ⋯*α*_*m*_ = *α*, 1/*q*_1_ + ⋯1/*q*_*m*_ = 1/*q*, 1/*p*_1_ + ⋯1/*p*_*m*_ = 1/*p*, *i* = 1,2,…, *m*. Then *ℋ*_*ω*_^*m*^ is bounded from ${\dot{K}}_{q_{1}}^{\alpha_{1},p_{1}}\times \cdots \times {\dot{K}}_{q_{m}}^{\alpha_{m},p_{m}}$ to ${\dot{K}}_{q}^{\alpha ,p}$ if

(18) $$\begin{array}{c} \int_{0<t_{1},\ldots ,t_{m}<1}\left(\overset{m}{\underset{i=1}{\prod }}t_{i}^{-(\alpha_{i}+n/q_{i})}\right)\omega \left(\vec{t}\right)d\vec{t}<\infty . \end{array}$$

Conversely, if *α*_1_ = ⋯ = *α*_*m*_ = (1/*m*)*α*, *q*_*i*_ = *mq*, *p*_*i*_ = *mp*, *i* = 1,2,…, *m*, and *ℋ*_*ω*_^*m*^ is bounded from ${\dot{K}}_{q_{1}}^{\alpha_{1},p_{1}}\times \cdots \times {\dot{K}}_{q_{m}}^{\alpha_{m},p_{m}}$ to ${\dot{K}}_{q}^{\alpha ,p}$, then (18) holds. Moreover, in this case, one has

(19) ||ℋωm||K˙q1α1,p1×⋯×K˙qmαm,pm→K˙qα,p ≃∫0<t1,…,tm<1(∏i=1mti−(αi+n/qi))ω(t→)dt→.

Similarly, we have the following result for the operator *𝒢*_*ω*_^*m*^.

**Theorem 6**

Let *α*, *α*_1_,…, *α*_2_ ∈ ℝ, 1 < *p*, *p*_*i*_, *q*, *q*_*i*_ < *∞*, and *α*_1_ + ⋯*α*_*m*_ = *α*, 1/*q*_1_ + ⋯1/*q*_*m*_ = 1/*q*, 1/*p*_1_ + ⋯1/*p*_*m*_ = 1/*p*, *i* = 1,2,…, *m*. If *ω* satisfies

(20) $$\begin{array}{c} \int_{0<t_{1},\ldots ,t_{m}<1}\left(\overset{m}{\underset{i=1}{\prod }}t_{i}^{\alpha_{i}-n(1-1/q_{i})}\right)\omega \left(\vec{t}\right)d\vec{t}<\infty , \end{array}$$

then *𝒢*_*ω*_^*m*^ is bounded from ${\dot{K}}_{q_{1}}^{\alpha_{1},p_{1}}\times \cdots \times {\dot{K}}_{q_{m}}^{\alpha_{m},p_{m}}$ to ${\dot{K}}_{q}^{\alpha ,p}$.

Conversely, if *α*_1_ = ⋯ = *α*_*m*_ = (1/*m*)*α*, *q*_*i*_ = *mq*, *p*_*i*_ = *mp*, *i* = 1,2,…, *m*, and *𝒢*_*ω*_^*m*^ is bounded from ${\dot{K}}_{q_{1}}^{\alpha_{1},p_{1}}\times \cdots \times {\dot{K}}_{q_{m}}^{\alpha_{m},p_{m}}$ to ${\dot{K}}_{q}^{\alpha ,p}$, then (20) holds. Moreover, in this case, one has

(21) ||𝒢ωm||K˙q1α1,p1×⋯×K˙qmαm,pm→K˙qα,p  ≃∫0<t1,…,tm<1(∏i=1mtiαi−n(1−1/qi))ω(t→)dt→.

Since the proofs of Theorems 5 and 6 are similar, we just give the proof of Theorem 5.

**Proof of Theorem 5**

In order to simplify the proof, we only consider the case of *m* = 2. Actually, a similar procedure works for all *m* ∈ *ℕ*.

Since 1/*q*_1_ + 1/*q*_2_ = 1/*q*, by Hölder and Minkowski inequality, we have

(22) ||ℋω2(f1,f2)χk||Lq(ℝn) =(∫Ck|∬01f1(t1x)f(t2x)ω(t1,t2)dt1dt2|q)1/q ≤∬01(∫Ck|f1(t1x)f(t2x)|q)1/qω(t1,t2)dt1dt2 ≤∬01(∫Ck|f1(t1x)|q1dx)1/q1    ×(∫Ck|f2(t2x)|q2dx)1/q2ω(t1,t2)dt1dt2.

For the arbitrary *t*_1_, *t*_2_ ∈ (0,1), we can find *m*, *l* ∈ *𝒵*, such that 2^*m*−1^ < *t*_1_ ≤ 2^*m*^ and 2^*l*−1^ < *t*_2_ ≤ 2^*l*^. By Minkowski inequality, we have

(23) ∬01(∫Ck|f1(t1x)|q1dx)1/q1  ×(∫Ck|f2(t2x)|q2dx)1/q2ω(t1,t2)dt1dt2≤∬01(∫t1Ck|f1(x)|q1dx)1/q1   ×(∫t2Ck|f2(x)|q2dx)1/q2t1−n/q1t2−n/q2ω(t1,t2)dt1dt2≤∬01(∫2k+m−2<|x|≤2k+m|f1(x)|q1dx)1/q1   ×(∫2k+l−2<|x|≤2k+l|f2(x)|q2dx)1/q2   ×t1−n/q1t2−n/q2ω(t1,t2)dt1dt2≤∬01(||χk+m−1||Lq1+||f1χk+m||Lq1)   ×(||f2χk+l−1||Lq2+||f2χk+l||Lq2)t1−n/q1   ×t2−n/q2ω(t1,t2)dt1dt2.

For 1/*p*_1_ + 1/*p*_2_ = 1/*p* and *α*_1_ + *α*_2_ = *α*, Hölder inequality and Minkowski inequality give

(24) ||ℋω2(f1,f2)||K˙qα,p=(∑k=−∞∞2kαp||ℋω2(f1,f2)χk||Lq(ℝn)p)1/p≤(∑k=−∞∞2kαp(∬01(||f1χk+m−1||Lq1+||f1χk+m||Lq1)        ×(||f2χk+l−1||Lq2+||f2χk+l||Lq2)        ×t1−n/q1t2−n/q2ω(t1,t2)dt1dt2)p)1/p≤∬01(∑k=−∞∞2kαp(||f1χk+m−1||Lq1+||f1χk+m||Lq1)p      ×(||f2χk+l−1||Lq2+||f2χk+l||Lq2)p)1/p     ×t1−n/q1t2−n/q2ω(t1,t2)dt1dt2≤∬01(∑k=−∞∞2kα1p1(||f1χk+m−1||Lq1         +||f1χk+m||Lq1)p1)1/p1   ×(∑k=−∞∞2kα2p2(||f2χk+l−1||Lq2+||f2χk+l||Lq2)p2)1/p2   ×t1−n/q1t2−n/q2ω(t1,t2)dt1dt2≤∬01{(∑k=−∞∞2kα1p1||f1χk+m−1||Lq1p1)1/p1    +(∑k=−∞∞2kα1p1||f1χk+m||Lq1p1)1/p1}   ×{(∑k=−∞∞2kα2p2||f2χk+l−1||Lq2p2)1/p2      +(∑k=−∞∞2kα2p2||f2χk+l||Lq2p2)1/p2}  ×t1−n/q1t2−n/q2ω(t1,t2)dt1dt2≤||f1||K˙q1α1,p1||f2||K˙q2α2,p2 ×∬01(2−(m−1)α1+2−mα1)(2−(l−1)α2+2−lα2)    ×t1−n/q1t2−n/q2ω(t1,t2)dt1dt2≤||f1||K˙q1α1,p1||f2||K˙q2α2,p2 ×∬01t1−(α1+n/q1)t2−(α2+n/q2)ω(t1,t2)dt1dt2.

From the above inequality, we have that the first conclusion in Theorem 5 holds.

On the other hand, we suppose that *ℋ*_*ω*_^2^ is bounded from ${\dot{K}}_{q_{1}}^{\alpha_{1},p_{1}}\times {\dot{K}}_{q_{2}}^{\alpha_{2},p_{2}}$ to ${\dot{K}}_{q}^{\alpha ,p}$ and *ℋ*_*ω*_^2^ has the operator norm ${||{ℋ}_{\omega }^{2}||}_{{\dot{K}}_{q_{1}}^{\alpha_{1},p_{1}}\times {\dot{K}}_{q_{2}}^{\alpha_{2},p_{2}}\to {\dot{K}}_{q}^{\alpha ,p}}$. For any 0 < *ε* < 1, let

(25) $$\begin{array}{c} f_{1}\left(x\right)=\{\begin{array}{cc} 0, & \left|x\right|\leq 1,\\ {\left|x\right|}^{-\alpha_{1}-n/q_{1}-\varepsilon }, & \left|x\right|>1, \end{array}\\ f_{2}\left(x\right)=\{\begin{array}{cc} 0, & \left|x\right|\leq 1,\\ {\left|x\right|}^{-\alpha_{2}-n/q_{2}-\varepsilon }, & \left|x\right|>1. \end{array} \end{array}$$

Obviously, ||*f*_1_ *χ*_*k*_||_*L*^*q*_1_^(ℝ^*n*^)_ = ||*f*_2_ *χ*_*k*_||_*L*^*q*_2_^(ℝ^*n*^)_ = 0, when *k* = 0, −1, −2,…. So, for any positive integer *k*, it is easy to get that

(26) ||f1χk||Lq1(ℝn)=(∫2k−1≤|x|<2k|x|(−α1−n/q1−ε)q1dx)1/q1=2−(α1+ε)k|Sn(2(α1+ε)q1−1)(α1+ε)q1|1/q1,

where *S*_*n*_ = *nπ* ^*n*/2^/Γ(1 + *n*/2). A simple computation gives

(27) ||f1||K˙q1α1,p1(ℝn) =2−ε(11−2−εp1)1/p1|Sn(2(α1+ε)q1−1)(α1+ε)q1|1/q1.

Similarly, we obtain

(28) ||f2||K˙q2α2,p2(ℝn) =2−ε(11−2−εp2)1/p2|Sn(2(α2+ε)q2−1)(α2+ε)q2|1/q2.

When |*x* | ≤1 and *t* ∈ [0,1], we have |*tx* | ≤1. So, *ℋ*_*ω*_^2^(*f*_1_, *f*_2_)(*x*) = 0 in this case based on (25). When |*x* | >1, we get

(29) ℋω2(f1,f2)(x)=∬1/|x|1f1(t1x)f2(t2x)ω(t1,t2)dt1dt2=|x|−(α+(n/q)+2ε)∬1/|x|1t1−α1−n/q1−εt2−α2−n/q2−ε          ×ω(t1,t2)dt1dt2.

Let *δ* = *ε* ^−1^ > 1. It is easy to find a positive integer *l* such that 2^*l*−1^ ≤ *δ* < 2^*l*^. Thus, we have

(30) ||ℋω2(f1,f2)||K˙qα,pp=∑k=1∞2kαp||ℋω2(f1,f2)χk(x)||Lq(ℝn)p=∑k=1∞2kαp{∫|x|>1(|x|−(α+n/q+2ε)χk(x)        ×∬1/|x|1t1−α1−n/q1−εt2−α2−n/q2−ε            ×ω(t1,t2)dt1dt2)qdx}p/q≥∑k=1∞2kαp(∫|x|>δ|x|−(α+n/q+2ε)qχk(x)dx)p/q ×(∬1/δ1t1−α1−n/q1−εt2−α2−n/q2−εω(t1,t2)dt1dt2)p≥Snp/q∑k=l+1∞2kαp(∫2k−12kr−(α+2ε)q−1dr)p/q ×(∬1/δ1t1−α1−n/q1−εt2−α2−n/q2−εω(t1,t2)dt1dt2)p=Snp/q∑k=l+1∞2−2εpk|(2(α+2ε)q−1)(α+2ε)q|p/q ×(∬1/δ1t1−α1−n/q1−εt2−α2−n/q2−εω(t1,t2)dt1dt2)p=Snp/q2−2ε(l+1)p1−2−2εp|(2(α+2ε)q−1)(α+2ε)q|p/q ×(∬1/δ1t1−α1−n/q1−εt2−α2−n/q2−εω(t1,t2)dt1dt2)p.

Since *q*_1_ = *q*_2_ = 2*q*, *α*_1_ = *α*_2_ = (1/2)*α* and 1/*p* = 1/*p*_1_ + 1/*p*_2_, *p*_1_ = *p*_2_ = 2*p*, we have

(31) ||ℋω2(f1,f2)||K˙qα,p ≥Sn1/q2−2ε(l+1)(11−2−2εp)1/p|(2(α+2ε)q−1)(α+2ε)q|1/q  ×∬1/δ1t1−α1−n/q1−εt2−α2−n/q2−εω(t1,t2)dt1dt2 ≥ε2ε||f1||K˙q1α1,p1||f2||K˙q2α2,p2  ×∬ε1t1−α1−n/q1−εt2−α2−n/q2−εω(t1,t2)dt1dt2.

This gives

(32) ||ℋω2(f1,f2)||K˙qα,p ≥ε2ε||f1||K˙q1α1,p1||f2||K˙q2α2,p2  ×∬ε1t1−α1−n/q1−εt2−α2−n/q2−εω(t1,t2)dt1dt2.

Using the boundedness of *ℋ*_*ω*_^2^ and its operator norm yields

(33) ε2ε∬ε1t1−α1−n/q1−εt2−α2−n/q2−εω(t1,t2)dt1dt2  ≤||ℋω2||K˙q1α1,p1×K˙q2α2,p2→K˙qα,p.

Letting *ε* → 0^+^ in (33) gives

(34) ∬01t1−α1−n/q1t2−α2−n/q2ω(t1,t2)dt1dt2  ≤||ℋω2||K˙q1α1,p1×K˙q2α2,p2→K˙qα,p.

Thus, (18) holds.

Since ${\dot{K}}_{p}^{0,p}({\mathbb{R}}^{n})=K_{p}^{0,p}({\mathbb{R}}^{n})=L^{p}({\mathbb{R}}^{n})$ and ${\dot{K}}_{p}^{\alpha /p,p}({\mathbb{R}}^{n})=K_{p}^{\alpha /p,p}({\mathbb{R}}^{n})=L^{p}(|x{|}^{\alpha }dx)$ for all 0 < *p* ≤ *∞*, we deduce the following corollaries from Theorems 5 and 6.

**Corollary 7**

Let *α*, *α*_1_,…, *α*_2_ ∈ ℝ, 1 < *p*, *p*_*i*_ < *∞* and *α*_1_ + ⋯*α*_*m*_ = *α*, 1/*p*_1_ + ⋯1/*p*_*m*_ = 1/*p*, *i* = 1,2,…, *m*. Then *ℋ*_*ω*_^*m*^ is bounded from *L* ^*p*_1_^(|*x*|^*α*_1_^ *dx*) × ⋯×*L* ^*p*_*m*_^(|*x*|^*α*_*m*_^ *dx*) to *L* ^*p*^(|*x*|^*α*^ *dx*) if

(35) $$\begin{array}{c} \int_{0<t_{1},\ldots ,t_{m}<1}\left(\overset{m}{\underset{i=1}{\prod }}t_{i}^{-(\alpha_{i}+n)/p_{i}}\right)\omega \left(\vec{t}\right)d\vec{t}<\infty . \end{array}$$

Conversely, if *α*_1_ = ⋯ = *α*_*m*_ = (1/*m*)*α*, *p*_*i*_ = *mp*, *i* = 1,2,…, *m*, and *ℋ*_*ω*_^*m*^ is bounded from *L* ^*p*_1_^(|*x*|^*α*_1_^ *dx*)×⋯×*L* ^*p*_*m*_^(|*x*|^*α*_*m*_^ *dx*) to *L* ^*p*^(|*x*|^*α*^ *dx*), then (35) holds. Moreover, in this case, one has

(36) ||ℋωm||Lp1(|x|α1dx)×⋯×Lpm(|x|αmdx)  →Lp(|x|αdx)   ≃∫0<t1,…,tm<1(∏i=1mti−(αi+n)/pi)ω(t→)dt→.

**Corollary 8**

Let *α*, *α*_1_,…, *α*_2_ ∈ ℝ, 1 < *p*, *p*_*i*_ < *∞* and *α*_1_ + ⋯*α*_*m*_ = *α*, 1/*p*_1_ + ⋯1/*p*_*m*_ = 1/*p*, *i* = 1, 2,…, *m*. If *ω* satisfies

(37) $$\begin{array}{c} \int_{0<t_{1},\ldots ,t_{m}<1}\left(\overset{m}{\underset{i=1}{\prod }}t_{i}^{\left(\alpha_{i}+n\right)/p_{i}-n}\right)\omega \left(\vec{t}\right)d\vec{t}<\infty , \end{array}$$

then *𝒢*_*ω*_^*m*^ is bounded from *L* ^*p*_1_^(|*x*|^*α*_1_^ *dx*)×⋯×*L* ^*p*_*m*_^(|*x*|^*α*_*m*_^ *dx*) to *L* ^*p*^(|*x*|^*α*^ *dx*).

Conversely, if *α*_1_ = ⋯ = *α*_*m*_ = (1/*m*)*α*, *p*_*i*_ = *mp*, *i* = 1,2,…, *m* and *𝒢*_*ω*_^*m*^ is bounded from *L* ^*p*_1_^(|*x*|^*α*_1_^ *dx*)×⋯×*L* ^*p*_*m*_^(|*x*|^*α*_*m*_^ *dx*) to *L* ^*p*^(|*x*|^*α*^ *dx*), then (37) holds. Moreover, in this case, one has

(38) ||𝒢ωm||Lp1(|x|α1dx)×⋯×Lpm(|x|αmdx)→Lp(|x|αdx) ≃∫0<t1,…,tm<1(∏i=1mti(αi+n)/pi−n)ω(t→)dt→.

## 3. Boundedness of *ℋ*_*ω*_^*m*^ on the Product of Morrey-Herz Spaces

Lets give the second main result of this paper.

**Theorem 9**

Let *α*, *α*_1_,…, *α*_2_ ∈ ℝ, *λ*, *λ*_*i*_ > 0, 1 < *p*, *p*_*i*_, *q*, *q*_*i*_ < *∞*, *i* = 1,2,…, *m*, and *α*_1_ + ⋯*α*_*m*_ = *α*, 1/*q*_1_ + ⋯+1/*q*_*m*_ = 1/*q*, 1/*p*_1_ + ⋯+1/*p*_*m*_ = 1/*p*, *λ*_1_ + ⋯+*λ*_*m*_ = *λ*. Then *ℋ*_*ω*_^*m*^ is bounded from $M{\dot{K}}_{p_{1},q_{1}}^{\alpha_{1},\lambda_{1}}\times \cdots \times M{\dot{K}}_{p_{m},q_{m}}^{\alpha_{m},\lambda_{m}}$ to $M{\dot{K}}_{p,q}^{\alpha ,\lambda }$ if *ω* satisfies

(39) $$\begin{array}{c} \int_{0<t_{1},\ldots ,t_{m}<1}\left(\overset{m}{\underset{i=1}{\prod }}t_{i}^{-(\alpha_{i}+n/q_{i}-\lambda_{i})}\right)\omega \left(\vec{t}\right)d\vec{t}<\infty . \end{array}$$

Conversely, when *α*_1_ = ⋯ = *α*_*m*_ = (1/*m*)*α*, *λ*_1_ = ⋯ = *λ*_*m*_ = (1/*m*)*λ*, *q*_*i*_ = *mq*, and *p*_*i*_ = *mp*, *i* = 1,2,…, *m*, if *ℋ*_*ω*_^*m*^ is bounded from $M{\dot{K}}_{p_{1},q_{1}}^{\alpha_{1},\lambda_{1}}\times \cdots \times M{\dot{K}}_{p_{m},q_{m}}^{\alpha_{m},\lambda_{m}}$ to $M{\dot{K}}_{p,q}^{\alpha ,\lambda }$, then (39) holds. And in this case, one has

(40) ||ℋωm||MK˙p1,q1α1,λ1×⋯×MK˙pm,qmαm,λm→MK˙p,qα,λ ≃∫0<t1,…,tm<1(∏i=1mti−(αi+n/qi−λi))ω(t→)dt→.

For the operator *𝒢*_*ω*_^*m*^, we have a corresponding result.

**Theorem 10**

Let *α*, *α*_1_,…, *α*_2_ ∈ ℝ, *λ*, *λ*_*i*_ > 0, 1 < *p*, *p*_*i*_, *q*, *q*_*i*_ < *∞*, *i* = 1,2,…, *m*, and *α*_1_ + ⋯+*α*_*m*_ = *α*, 1/*q*_1_ + ⋯+1/*q*_*m*_ = 1/*q*, 1/*p*_1_ + ⋯+1/*p*_*m*_ = 1/*p*, *λ*_1_ + ⋯+*λ*_*m*_ = *λ*. Then *𝒢*_*ω*_^*m*^ is bounded from $M{\dot{K}}_{p_{1},q_{1}}^{\alpha_{1},\lambda_{1}}\times \cdots \times M{\dot{K}}_{p_{m},q_{m}}^{\alpha_{m},\lambda_{m}}$ to $M{\dot{K}}_{p,q}^{\alpha ,\lambda }$ if *ω* satisfies

(41) $$\begin{array}{c} \int_{0<t_{1},\ldots ,t_{m}<1}\left(\overset{m}{\underset{i=1}{\prod }}t_{i}^{\alpha_{i}-\lambda_{i}-n(1-1/q_{i})}\right)\omega \left(\vec{t}\right)d\vec{t}<\infty . \end{array}$$

Conversely, when *α*_1_ = ⋯ = *α*_*m*_ = (1/*m*)*α*, *λ*_1_ = ⋯ = *λ*_*m*_ = (1/*m*)*λ*, *q*_*i*_ = *mq*, and *p*_*i*_ = *mp*, *i* = 1,2,…, *m*, if *𝒢*_*ω*_^*m*^ is bounded from $M{\dot{K}}_{p_{1},q_{1}}^{\alpha_{1},\lambda_{1}}\times \cdots \times M{\dot{K}}_{p_{m},q_{m}}^{\alpha_{m},\lambda_{m}}$ to $M{\dot{K}}_{p,q}^{\alpha ,\lambda }$, then (41) holds and

(42) ||ℋωm||MK˙p1,q1α1,λ1×⋯×MK˙pm,qmαm,λm→MK˙p,qα,λ  ≃∫0<t1,…,tm<1(∏i=1mtiαi−λi−n(1−1/qi))ω(t→)dt→.

Since the proof of Theorem 10 is similar to the proof of Theorem 9, we give the proof of Theorem 9 only.

**Proof of Theorem 9**

By similarity, we only consider the case that *m* = 2. From the proof of Theorem 5, we know that

(43) ||ℋω2(f1,f2)χk||Lq(ℝn) ≤∬01(||f1χk+m−1||Lq1+||f1χk+m||Lq1)    ×(||f2χk+l−1||Lq2+||f2χk+l||Lq2)    ×t1−n/q1t2−n/q2ω(t1,t2)dt1dt2.

For 1/*p* = 1/*p*_1_ + 1/*p*_2_ and *α* = *α*_1_ + *α*_2_, *λ* = *λ*_1_ + *λ*_2_, Hölder inequality and Minkowski inequality give

(44) ||ℋω2(f1,f2)||MK˙p,qα,λ=supk0∈ℤ2−k0λ(∑k=−∞k02kαp||ℋω2(f1,f2)χk||Lq(ℝn)p)1/p≤supk0∈ℤ2−k0λ(∑k=−∞k02kαp       ×(∬01(||f1χk+m−1||Lq1+||f1χk+m||Lq1)          ×(||f2χk+l−1||Lq2+||f2χk+l||Lq2)          ×t1−n/q1t2−n/q2ω(t1,t2)dt1dt2)p)1/p≤supk0∈ℤ2−k0λ∬01(∑k=−∞k02kαp(||f1χk+m−1||Lq1            +||f1χk+m||Lq1)p           ×(||f2χk+l−1||Lq2+||f2χk+l||Lq2)p)1/p        ×t1−n/q1t2−n/q2ω(t1,t2)dt1dt2≤supk0∈ℤ2−k0λ∬01(∑k=−∞k02kα1p1(||f1χk+m−1||Lq1            +||f1χk+m||Lq1)p1)1/p1      ×(∑k=−∞k02kα2p2(||f2χk+l−1||Lq2             +||f2χk+l||Lq2)p2)1/p2      ×t1−n/q1t2−n/q2ω(t1,t2)dt1dt2≤supk0∈ℤ2−k0λ1∬01{(∑k=−∞k02kα1p1||f1χk+m−1||Lq1p1)1/p1        +(∑k=−∞k02kα1p1||f1χk+m||Lq1p1)1/p1}       ×supk0∈ℤ2−k0λ2{(∑k=−∞k02kα2p2||f2χk+l−1||Lq2p2)1/p2           +(∑k=−∞k02kα2p2               ×||f2χk+l||Lq2p2)1/p2}       ×t1−n/q1t2−n/q2ω(t1,t2)dt1dt2≤||f1||MK˙p1,q1α1,λ1||f2||MK˙p2,q2α2,λ2 ×∬01(2−(m−1)α1+2−mα1)(2−(l−1)α2+2−lα2)    ×t1−(n/q1−λ1)t2−(n/q2−λ2)ω(t1,t2)dt1dt2≤||f1||MK˙p1,q1α1,λ1||f2||MK˙p2,q2α2,λ2 ×∬01t1−(α1+n/q1−λ1)t2−(α2+n/q2−λ2)ω(t1,t2)dt1dt2.

It means that *ℋ*_*ω*_^*m*^ is bounded from $M{\dot{K}}_{p_{1},q_{1}}^{\alpha_{1},\lambda_{1}}\times \cdots \times M{\dot{K}}_{p_{m},q_{m}}^{\alpha_{m},\lambda_{m}}$ to $M{\dot{K}}_{p,q}^{\alpha ,\lambda }$.

On the other hand, we define

(45) $$\begin{array}{c} f_{1}\left(x\right)={\left|x\right|}^{-(\alpha_{1}+n/q_{1}-\lambda_{1})},\;x\in {\mathbb{R}}^{n},\\ f_{2}\left(x\right)={\left|x\right|}^{-(\alpha_{2}+n/q_{2}-\lambda_{2})},\;x\in {\mathbb{R}}^{n}. \end{array}$$

When *α*_1_ ≠ *λ*_1_ and *α*_2_ ≠ *λ*_2_, we get

(46) ||f1χk||Lq1=(∫2k−1≤|x|<2k|x|−(α1+n/q1−λ1)q1dx)1/q1=2−(α1−λ1)k|Sn(2(α1−λ1)q1−1)(α1−λ1)q1|1/q1,

where *S*_*n*_ = *nπ* ^*n*/2^/Γ(1 + *n*/2). Thus

(47) ||f1||MK˙p1,q1α1,λ1  =2λ1(12λ1p1−1)1/p1|Sn(2(α1−λ1)q1−1)(α1−λ1)q1|1/q1.

It is similar to obtain

(48) ||f2||MK˙p2,q2α2,λ2  =2λ2(12λ2p2−1)1/p2|Sn(2(α2−λ2)q2−1)(α2−λ2)q2|1/q2.

For *α* = *α*_1_ + *α*_2_, *λ* = *λ*_1_ + *λ*_2_, and 1/*q* = 1/*q*_1_ + 1/*q*_2_, we have

(49) ℋω2(f1,f2)(x)=|x|−(α+n/q−λ)∬01t1−(α1+n/q1−λ1)t2−(α2+n/q2−λ2)         ×ω(t1,t2)dt1dt2.

By simple computation, we get

(50) ||ℋω2(f1,f2)χk||Lq(ℝn)p=(∫ℝn|ℋω2(f1,f2)(x)|qχk(x)dx)p/q=(∫ℝn|x|−(α+n/q−λ)qχk(x)   ×(∬01t1−(α1+n/q1−λ1)t2−(α2+n/q2−λ2)       ×ω(t1,t2)dt1dt2)qdx)p/q=(∫2k−1≤|x|<2k|x|−(α+n/q−λ)qdx)p/q ×(∬01t1−(α1+n/q1−λ1)t2−(α2+n/q2−λ2)     ×ω(t1,t2)dt1dt2)p=2−(α−λ)kp|Sn(2(α−λ)q−1)(α−λ)q|p/q ×(∬01t1−(α1+n/q1−λ1)t2−(α2+n/q2−λ2)     ×ω(t1,t2)dt1dt2)p.

Since *α*_1_ = *α*_2_ = (1/2)*α*, *p*_1_ = *p*_2_ = 2*p*, *q*_1_ = *q*_2_ = 2*q*, and *λ*_1_ = *λ*_2_ = (1/2)*λ*, we have

(51) ||ℋω2(f1,f2)||MK˙p,qα,λ=supk0∈ℤ2−k0λ(∑k=−∞k02kαp||ℋω2(f1,f2)χk||Lq(ℝn)p)1/p=supk0∈ℤ2−k0λ(∑k=−∞k02kαp2−(α−λ)kp)1/p ×|Sn(2(α−λ)q−1)(α−λ)q|1/q ×∬01t1−(α1+n/q1−λ1)t2−(α2+n/q2−λ2)    ×ω(t1,t2)dt1dt2=2λ(12λp−1)1/p|Sn(2(α−λ)q−1)(α−λ)q|1/q ×∬01t1−(α1+n/q1−λ1)t2−(α2+n/q2−λ2)    ×ω(t1,t2)dt1dt2=||f1||MK˙p1,q1α1,λ1||f2||MK˙p2,q2α2,λ2 ×∬01t1−(α1+n/q1−λ1)t2−(α2+n/q2−λ2)    ×ω(t1,t2)dt1dt2.

Thus, (39) holds in this case.

When *α*_1_ = *λ*_1_ and *α*_2_ = *λ*_2_, it is easy to obtain

(52) ||f1χk||Lq1q1=||f2χk||Lq2q2=∫2k−1≤|x|<2k|x|−ndx=Snln2.

Thus, we have

(53) $$\begin{array}{c} {||f_{1}||}_{M{\dot{K}}_{p_{1},q_{1}}^{\alpha_{1},\lambda_{1}}}=2^{\lambda_{1}}{\left(\frac{1}{2^{\lambda_{1}p_{1}}-1}\right)}^{1/p_{1}}{\left(S_{n}\ln2\right)}^{1/q_{1}},\\ {||f_{2}||}_{M{\dot{K}}_{p_{2},q_{2}}^{\alpha_{2},\lambda_{2}}}=2^{\lambda_{2}}{\left(\frac{1}{2^{\lambda_{2}p_{2}}-1}\right)}^{1/p_{2}}{\left(S_{n}\ln2\right)}^{1/q_{2}}. \end{array}$$

So

(54) ℋω2(f1,f2)(x) =|x|−n/q∬01t1−n/q1t2−n/q2ω(t1,t2)dt1dt2.

Then, we get

(55) ||ℋω2(f1,f2)χk||Lq(ℝn) =(Snln2)1/q∬01t1−n/q1t2−n/q2ω(t1,t2)dt1dt2.

Thus,

(56) ||ℋω2(f1,f2)||MK˙p,qα,λ=supk0∈ℤ2−k0λ(∑k=−∞k02kαp||ℋω2(f1,f2)χk||Lq(ℝn)p)1/p=supk0∈ℤ2−k0λ(∑k=−∞k02kαp(Snln2)p/q       ×(∬01t1−n/q1t2−n/q2          ×ω(t1,t2)dt1dt2)p)1/p=supk0∈ℤ2−k0λ(∑k=−∞k02kαp)1/p(Snln2)1/q ×∬01t1−n/q1t2−n/q2ω(t1,t2)dt1dt2.

Since *α*_1_ = *λ*_1_ and *α*_2_ = *λ*_2_, we get *α* = *λ*. Thus

(57) ||ℋω2(f1,f2)||MK˙p,qα,λ  =2λ(12λp−1)1/p(Snln2)1/q   ×∬01t1−n/q1t2−n/q2ω(t1,t2)dt1dt2  =||f1||MK˙p1,q1α1,λ1||f2||MK˙p2,q2α2,λ2   ×∬01t1−n/q1t2−n/q2ω(t1,t2)dt1dt2.

This tells us that (39) also holds in this case.

Furthermore, when one of *α*_1_ = *λ*_1_, *α*_2_ = *λ*_2_ holds, we suppose *α*_1_ = *λ*_1_ but *α*_2_ ≠ *λ*_2_ here. From the above computation, we have

(58) ||f1||MK˙p1,q1α1,λ1=2λ1(12λ1p1−1)1/p1(Snln2)1/q1,||f2||MK˙p2,q2α2,λ2  =2λ2(12λ2p2−1)1/p2|Sn(2(α2−λ2)q2−1)(α2−λ2)q2|1/q2.

It gives

(59) ℋω2(f1,f2)(x) =|x|−(α2+(n/q)−λ2)∬01t1−n/q1t2−(α2+n/q2−λ2)          ×ω(t1,t2)dt1dt2.

So, we have

(60) ||ℋω2(f1,f2)||MK˙p,qα,λ=supk0∈ℤ2−k0λ(∑k=−∞k02kαp||ℋω2(f1,f2)χk||Lq(ℝn)p)1/p=supk0∈ℤ2−k0λ(∑k=−∞k02kαp2−(α2−λ2)kp)1/p ×|Sn(2(α2−λ2)q−1)(α2−λ2)q|1/q ×∬01t1−n/q1t2−(α2+n/q2−λ2)ω(t1,t2)dt1dt2=2λ(12λp−1)1/p|Sn(2(α2−λ2)q−1)(α2−λ2)q|1/q ×∬01t1−n/q1t2−(α2+n/q2−λ2)ω(t1,t2)dt1dt2.

Since *α*_1_ = *α*_2_ = (1/2)*α*, *p*_1_ = *p*_2_ = 2*p*, *q*_1_ = *q*_2_ = 2*q*, and *λ*_1_ = *λ*_2_ = (1/2)*λ*, using Taylor expansion yields

(61) |Sn(2(α2−λ2)q−1)(α2−λ2)q|1/q  ≥(Snln2)1/q1|Sn(2(α2−λ2)q2−1)(α2−λ2)q2|1/q2.

Thus, we have

(62) ||ℋω2(f1,f2)||MK˙p,qα,λ ≥||f1||MK˙p1,q1α1,λ1||f2||MK˙p2,q2α2,λ2  ×∬01t1−n/q1t2−(α2+n/q2−λ2)ω(t1,t2)dt1dt2.

The above inequality gives

(63) ||ℋωm||MK˙p1,q1α1,λ1×⋯×MK˙pm,qmαm,λm→MK˙p,qα,λ  ≥∫0<t1,…,tm<1(∏i=1mti−(αi+n/qi−λi))ω(t→)dt→.

Since *ℋ*_*ω*_^*m*^ is bounded from $M{\dot{K}}_{p_{1},q_{1}}^{\alpha_{1},\lambda_{1}}\times \cdots \times M{\dot{K}}_{p_{m},q_{m}}^{\alpha_{m},\lambda_{m}}$ to $M{\dot{K}}_{p,q}^{\alpha ,\lambda }$, we know that (39) holds and

(64) ||ℋωm||MK˙p1,q1α1,λ1×⋯×MK˙pm,qmαm,λm→MK˙p,qα,λ  ≃∫0<t1,…,tm<1(∏i=1mti−(αi+n/qi−λi))ω(t→)dt→.

The proof of Theorem 9 is complete.
